# Environmental stress, minority status, and local poverty: risk factors for mental health in Berlin’s inner city

**DOI:** 10.1007/s00406-022-01508-3

**Published:** 2022-11-05

**Authors:** Debora Darabi, Ulrike Kluge, Simone Penka, Adrian P. Mundt, Meryam Schouler-Ocak, Jeffrey Butler, Shuyan Liu, Andreas Heinz, Michael A. Rapp

**Affiliations:** 1 Department of Psychiatry and Psychotherapy, Campus Mitte, Charité University Medicine Berlin, Berlin, Germany; 2 Social and Preventive Medicine, University of Potsdam, Potsdam, Germany; 3 Berlin Institute for Integration and Migration Research, Humboldt University, Berlin, Germany; 4 Departamento de Psiquiatría y Salud Mental, Hospital Clíınico Universidad de Chile, Santiago, Chile; 5 Facultad de Medicina, Universidad Diego Portales, Santiago, Chile; 6 Institute for Geography, Humboldt University, Berlin, Germany

**Keywords:** Local poverty, Mental health, Environmental stressors, Climate change, Social justice

## Abstract

This study examines whether climate change-associated environmental stressors, including air and noise pollution, local heat levels, as well as a lack of surrounding greenspace, mediate the effects of local poverty on mental health, using the 28-item General Health Questionnaire. We recruited 478 adults who were representative of eleven of Berlin’s inner-city neighborhoods. The relationship of individual-level variables, neighborhood-level sociodemographic and environmental data from the Berlin Senate (Department for Urban Development, Building and Housing) to mental health was assessed in a multilevel model using SPSS. We found that neither local exposure to environmental stressors, nor available greenspace as a protective factor, mediated the effects of local poverty on variance in mental health (all *p* values > 0.2). However, surrounding greenspace (*r* = -0.24, *p* < 0.001), nitrogen dioxide levels (*r* = 0.10, *p* < 0.05), noise pollution (rho = 0.15, *p* < 0.01), and particle pollution (*r* = 0.12, *p* < 0.001) were associated with local poverty, which, more strongly than individual factors, accounted for variance in mental health (*β* = 0.47, *p* < 0.001). Our analysis indicates that the effects of local poverty on mental health are not mediated by environmental factors. Instead, local poverty was associated with both an increased mental health burden and the exposure to climate-related environmental stressors.

## Introduction

Climate change is the major public health challenge of our time and threatens life on this planet [1]. Globally, vulnerable populations, such as poor communities or racialized minority groups, are disproportionately exposed to the increasing environmental stress that accompanies climate change [2, 3]. Meanwhile, these populations have little or no responsibility for causing climate change [4, 5] and often do not have the necessary financial and psychosocial resources to cope with its disastrous consequences [6].

Several epidemiological publications suggest that climate change is not only correlated with a well-documented rise in somatic disorders, i.e., respiratory diseases [7]. Long-term exposure to climate change-related environmental stressors, such as hot weather or air and noise pollution, is also associated with a greater mental health burden [8–14]. Further studies have examined whether surrounding greenspace acts as a protective factor, while a lack of available surrounding greenspace increases the risk of mental health-associated morbidity and mortality [15–19].

Importantly, socioeconomic inequality has been shown to mediate the effects of climate change-associated heat exposure on mental health-related hospitalizations. A nationwide case-crossover study in Brazil found that low-income communities exhibited a greater increase in mental health conditions than high-income communities when exposed to the same rise in temperature associated with climate change [20].

Our previous study showed that local poverty in the neighborhood is related to an increased mental health burden when controlling for individual income and years of education [21]. Here, we aim to broaden our research by measuring associations between local poverty and environmental factors and assessing whether the effects of local poverty on mental health are mediated by local heat exposure [22], air pollution [23], noise pollution [24], and greenspace exposure [25].

## Materials and methods

### Sample

We used Berlin neighborhood-level sociodemographic and environmental variables gathered by the Berlin Senate (Department for Urban Development, Building and Housing) and the Berlin-Brandenburg Office for Statistics from 2009 to 2011. They included age and gender distribution, ethnic density, and local poverty, defined as the percentage of social welfare (Arbeitslosengeld II) recipients in each neighborhood. We also obtained five indicators of environmental stress for each neighborhood in the same period of time—the average annual external cost of continued disturbance and health risks due to noise-related stress per person (low = 0–21€ and high = 40–103€), surrounding green space (m^2^), physiological equivalent temperature (°C), air pollution as indexed by the amount of nitrogen dioxide (NO_2_) and the average fine particulate matter (PM_2.5_) in the atmosphere.

In its Environmental Justice Atlas, the Berlin Senate Administration for Urban Development, Building and Housing examined the effects of several environmental stress factors at the neighborhood level [26]. In this context, the external cost of noise-related stress was modeled by creating a complete 3D-model for all of Berlin, with a population of 3.460.725 in 2010, in IMMI 2012, a software tool for calculating environmental noise [27]. This model consisted of sub-models for different sources including railroads, bridges, embankments, tunnels, all forms of public transportation, road traffic, industrial sites, and air traffic. To show the impact of noise pollution, the average annual external costs of the resulting health burden per person in a given location and a cost stratification classifying noise pollution from low-impact, low-cost to high-impact, high-cost noise-related stress were calculated [27]. The available public green space was measured using the green space information system (GRIS) [28]. The physiological equivalent temperature (PET), a measure of heat stress combining atmospheric temperature with latent heat [29], was calculated using the Flow over Irregular Terrain with Natural and Anthropogenic Heat sources (FITNAH) climate model, merged with data from long-standing measuring stations in Berlin and Potsdam for baseline temperature values [30]. By averaging the daily data from 400 detectors in 300 different locations throughout Berlin’s main street grid counting the number of vehicles, differentiating between smaller and larger vehicles, and calculating the yearly proportional average for all of Berlin’s different neighborhoods, NO_2_ and PM_2,5_ emissions were calculated [31]. The environmental impact of public transportation was accounted for by factoring in location-specific public bus schedules and for motorbikes by merging the count with data from a proportionally adjusted manual count from 2009. The average emissions for all forms of transportation combined were calculated in IMMIS [32], using the Federal Environmental Agency’s handbook for emission factors [33].

We matched the local-level data with subjective, individual-level parameters. We included respondents with (*n* = 204) and without (*n* = 274) a migration background (total *n* = 478) selected from public registries of all residents in eleven neighborhoods in Berlin’s inner city (Berlin-Mitte), combined with on-site selection and snowballing, interviewed in 2009–2011 [34]. Our sample reflects the average age and gender distribution of the respective neighborhood according to local registry data [35]. To further assess epidemiological representativeness, we tested our respondents’ data against data from a representative sample of the local neighborhood [36] and did not find significant differences. Participants were contacted up to three times in writing or by telephone. Interviews were conducted in German and Turkish by trained interviewers and comprised a sociodemographic assessment and the General Health Questionnaire 28-item version (GHQ-28) [37, 38], with ratings ranging from 0 to 3, thus yielding a possible total score of 84. A cutoff score of 23/24 has been suggested as indicative of significant distress [37, 38].

### Data analysis

In the present analyses, we first explored direct associations of the neighborhood-level environmental variables (greenspace, noise pollution, nitrogen dioxide levels, particle pollution, and physiological equivalent temperature) and local poverty with mental health in a two-tailed correlation test. We then modeled the following variables: age, gender, years of total schooling, monthly net income per household member, and minority status at the individual and the neighborhood level, as well as environmental variables at the neighborhood level, to examine whether the latter mediate the effects of local poverty on mental health in a multilevel mediation model using SPSS (IBM, 2022).

For the statistical analysis at the neighborhood level, we combined the thirteen planning areas that make up Moabit, as one of the eleven neighborhoods in our study, since we did not have enough respondents for each of the individual planning areas. We then calculated a weighted average for each of our environmental variables for Moabit as one unified neighborhood. Finally, for the precision of our multilevel model, we calculated a weighted average for noise pollution, which was originally coded on an ordinal scale, instead of a mode, which would be less accurate.

## Results

This study included *n* = 478 adults living in eleven of Berlin’s inner-city neighborhoods. Participants aged from 18 to 68 years were included. Women represented 51% of the sample. Participants with a migration background made up 43% of our sample. An overview over further sociodemographic factors as well as mental health variables is provided in Table 1.

**Table 1:** Descriptive statistics and study variables

Variables	Valid cases	Minimum	Maximum	Mean	Standard deviation	Variance
Demographic factors						
Gender	478					
Male	234			49%^P^		
Female	244			51%^p^		
Age	478	18	68	41.55	11.47	131.49
Migration background	478					
Yes	204			43%^P^		
No	274			57%^P^		
Education level (years of formal education)	478	0	15	8.59	4.42	19.51
Individual income (€/month)	478	100	1600	470.92	262.97	69,152.61
Mental health^GHQ^						
Individual	478	0	82	19.28	18.41	338.84
Neighborhood-level^N^						
Moabit (2100)	31	0	30	12.1	9.51	90.49
Soldiner Str. (3100)	38	5	82	26.26	19.89	395.5
Gesundbrunnen (3102)	23	0	71	21	18.99	360.73
Brunnenstr. (3200)	27	0	52	19.52	15.12	228.64
Humboldthain Nordwest (3203)	38	0	76	21.05	20.46	418.54
Rehberge (4101)	48	0	63	18.52	18.71	350.13
Schillerpark (4102)	35	0	54	13.03	14.02	196.62
Westliche Müllerstr. (4103)	26	0	56	10.69	13.68	187.18
Reinickendorfer Str. (4201)	81	3	82	23.05	22.09	487.85
Sparrplatz (4202)	51	0	72	18.75	18.14	329.19
Leopoldplatz (4203)	80	3	66	19.84	17.23	296.92

*P* percentage of total sample, *GHQ* mental health according to the GHQ-28 with scores ranging from 0 to 84, *N* statistical planning areas defined by the Berlin Senate were used to delineate neighborhoods

At the neighborhood level, greenspace (*r* = − 0.24, *p* < 0.001), nitrogen dioxide levels (*r* = 0.10, *p* < 0.05), particle pollution (*r* = 0.12, *p* < 0.01), and noise pollution (rho = 0.15, *p* < 0.01) were associated with local poverty, defined as the percentage of citizens on social welfare in the respective neighborhood. Temperature, on the other hand, was not significantly associated with local poverty (Table 2).

**Table 2:** Correlations (Pearson *r*, Spearman rho, and two-tailed *P* value) among the neighborhood variables

Variable	Greenspace	Nitrogen dioxide levels	Particle pollution	Noise pollution	Equivalent temperature
Local poverty	*r* = − 0.24***	*r* = 0.10*	*r* = 0.12**	rho = 0.15**	*r* = 0.04^ns^

***p < 0.001, **p < 0.01, *p < 0.05, *ns* not significant

When adding both local sociodemographic and environmental neighborhood effects as well as individual effects in a multilevel model, local poverty (*β* = 0.47, *p* < 0.001) and, to a lesser degree, older age (*β* = 0.15, *p* < 0.05) accounted for variance in mental health. The effect of individual poverty was no longer significant (*β* = − 0.81, *p* = 0.051), and we did not find significant effects of environmental neighborhood variables on mental health (all *p* values > 0.2, Table 3).

**Table 3:** Multilevel model for effects of environmental variables, local poverty, and sociodemographic variables on mental health

Parameter	Beta	Standard Error	*df*	*t*	Significance	95% confidence interval	
						Lower bound	Upper bound
Age	0.147846	0.070527	466	2.096	0.037	0.09256	0.286436
Gender	− 2.903499	1.669457	466	− 1.739	0.083	− 6.184095	0.377098
Education level	0.009315	0.246708	466	0.038	0.970	− 0.475483	0.494113
Individual income	− 0.008052	0.004122	466	− 1.954	0.051	− 0.016151	4.753003E−5
Minority status	3.456637	1.762801	466	1.961	0.050	− 0.007386	6.920659
Greenspace	− 2.284375E−7	4.809375E−6	466	− 0.047	0.962	− 9.679186E−6	9.222311E−6
Noise pollution	− 0.099874	1.961886	466	− 0.051	0.959	− 3.955114	3.755365
Nitrogen dioxide levels	0.685259	1.692213	466	0.405	0.686	− 2.640054	4.010571
Average atmospheric fine particulate matter (PM_2.5_)	− 1.508206	2.866983	466	− 0.526	0.599	− 7.142022	4.125611
Equivalent temperature	− 0.342753	1.370485	466	− 0.250	0.803	− 3.035849	2.350344
Local poverty	0.466385	0.111074	466	4.199	< 0.001	0.248116	0.684653

Dependent Variable: mental health

The mediation analysis assessing whether effects of local poverty on mental health were mediated by environmental stressors, did not yield any significant associations between environmental variables and the outcome mental health, thus violating mediation analysis assumptions. Neither were there any significant cross-level interactions between individual sociodemographic data, minority status and environmental data (all *p* values > 0.23). We therefore refrained from calculating path coefficients.

## Discussion

To the best of our knowledge, this is the first study that simultaneously assesses the impact of (1) local environmental stressors (heat, air and noise pollution) [22–24] and potential resilience factors such as greenspace exposure [25], as well as (2) neighborhood data on local poverty, and (3) data at the individual level on mental health outcomes.

Consistent with another German study on the association between air pollution and mental well-being [39], which found no association between anxiety or depression and air pollution, our results highlight that local heat exposure, as well as noise and air pollution, were not independently associated with an increased mental health burden. Similarly, local greenspace did not act as a protective factor against the adverse effects of local poverty on mental health in our study. Finally, none of the environmental variables mediated the effects of local poverty on mental health. However, we found that local poverty was associated with a higher mental health burden as well as increased local exposure to environmental stress. Local poverty thus increases vulnerability for both a high mental health burden as well as for environmental stress potentially associated with climate change—including noise and air pollution or lack of greenspaces.

Several limitations should be addressed. The data in this study is from 2011 and shows an association between local poverty, environmental stress, and an increased mental health burden, prior to significant developments such as the 2015 European migrant crisis, the COVID-19 pandemic, the Russian invasion of Ukraine and its global economic consequences, as well as the increasing global awareness of the climate crisis. These changing circumstances will have affected the urban landscape and the associations described in this study. Therefore, further continuous research on the association between local poverty, environmental stress, and mental health is essential to adequately analyze constantly evolving social relations. This paper looks to provide a foundation for such pivotal future research.

Altogether, our data emphasizes the effect of local poverty on mental health. Our previous studies showed that structurally discriminated minorities were particularly vulnerable to local poverty, potentially due to social exclusion and discrimination [21, 40]. Future studies focusing on larger regional and environmental differences between communities should include a specific assessment of minority status as a potential risk factor. Regarding possible public health interventions, our findings suggest that preventive mental health strategies in urban populations need to be multi-factorial. Accordingly, public health strategies seeking to mitigate the adverse effects of environmental stress on mental health disorders without addressing local poverty or social exclusion could fall short, since environmental stress in our study was not associated with mental health independently from local poverty. Policies addressing local poverty could thus be combined with urban planning to alleviate the significant burden of increased environmental stress, to which residents of poor neighborhoods are particularly exposed [20, 40].

## Data Availability

The data that support the findings of this study are available from the corresponding author, D.D., upon reasonable request.
